# A Novel Approach to Posterior Lateral Nasal Neurectomy

**DOI:** 10.7759/cureus.39973

**Published:** 2023-06-05

**Authors:** Sanjana K Reddy, K. C Prasad, Kouser Mohammadi, Lini Joseph, Rohitha Meenavalli

**Affiliations:** 1 Otolaryngology, Sri Devaraj Urs Medical College, Kolar, IND

**Keywords:** india, allergic rhinitis, hemoglobin, posterior nasal nerve neurectomy, posterior lateral nasal neurectomy

## Abstract

Background

Allergic rhinitis (AR) is a major health concern throughout the world. By severing the parasympathetic supply to the lateral wall of the nose, posterior lateral nasal neurectomy (PLNN), a form of highly selective vidian neurectomy, decreases nasal allergy symptoms. This study attempts to characterize the demographic and surgical characteristics of study participants in relation to PLNN, as well as to identify the risk factors associated with these characteristics.

Methodology

A five-year, cross-sectional study was undertaken among patients diagnosed with AR at a tertiary care center in Tamaka, Kolar. Case sheets accessible in the department of medical records were used to compile a list of 50 study patients. SPSS version 21 was used for data analysis (IBM Corp., Armonk, NY, USA).

Results

The study revealed that the average age of the sample population was 30.4 years. The majority of the study participants were less than or equal to 30 years old (54%). In our study, the majority of the participants were male (60%). This study revealed that around 46% of the surgeries were independent PLNNs and that most of them (76%) were observed to have four nerves following surgery. The average intraoperative blood loss during PLNN surgery was 43.14 mL. The mean hemoglobin levels before and after surgery were 13.11 and 12.78 g/dL, respectively. The average duration of the surgical procedure was 62 minutes. The average duration of PLNN surgery in females was 52.75 minutes, whereas the average duration in males was 68.33 minutes. According to an independent t-test (p = 0.045), this difference in mean was statistically significant. Approximately 85% of female study participants were identified with four nerves during PLNN surgery compared to 70% of male study participants. According to the chi-square test (p = 0.018), this proportional difference was statistically significant.

Conclusions

The majority of the participants in this study were male and younger. The typical PLNN surgical procedure lasted one hour. Males and females require different amounts of time, with females requiring less time. During PLNN surgery, most females detected four nerves, as opposed to most males.

## Introduction

Allergic rhinitis (AR) is characterized by nasal mucosal inflammation in reaction to allergens, resulting in symptoms such as nasal congestion and pruritis, non-infectious sneezing, and watery rhinorrhea [1]. The global spread of AR is a major public health concern. More than 500 million people reported experiencing AR symptoms, and their frequency has grown over the past few decades [1,2].

A subset of individuals with AR may also have eye issues, a chronic cough, and postnasal drip, which gravely diminish their quality of life. Oral antihistamines and intranasal corticosteroids are common first-line therapies for AR [3,4].

Subcutaneous or sublingual allergen-specific immunotherapy can frequently reduce symptoms in patients who do not exhibit an adequate response to standard treatment. A small minority of patients are resistant to these conservative treatments, and immunotherapy can cause devastating adverse effects such as anaphylaxis. The management techniques vary mainly based on instrumentation and the avoidance of postoperative epistaxis, transient hypoesthesia of the soft palate, and eye dryness. Endoscopic visualization and cauterization or resection of posterior nasal nerve branches can prevent such complications [5]. Surgical treatment options for these patients include vidian neurectomy, inferior turbinate reduction, and the more modern posterior lateral nasal neurectomy (PLNN), which involves selective transection of vidian nerve branches [5].

The posterior nasal nerve is composed of sensory fibers from the maxillary nerve and the postganglionic pterygoid nerve. It emerges from the sphenopalatine ganglion and enters the nasal cavity through the sphenopalatine foramen [6,7]. By severing the parasympathetic supply to the lateral wall of the nose, PLNN, a form of highly selective vidian neurectomy, decreases symptoms of nasal allergy. In addition, PLNN minimizes the likelihood of developing long-term side effects, such as palate numbness and persistent dry eyes, after a vidian neurectomy [8,9].

The nasopharyngeal mucosa is innervated by the pharyngeal nerve, a branch of the pterygopalatine ganglion conveyed by the palatovaginal canal, and adjacent to the region of the nasal mucosa controlled by the posterior nasal nerve [10]. Nasal obstruction and rhinorrhea both markedly improve after posterior nasal nerve resection. While there is no discernible change in the density of the arteries, morphometric analysis of the nasal gland density reveals a considerable reduction. It is observed that the amount of infiltrating neutrophils, eosinophils, and lymphocytes significantly decreases [11]. In the existing literature, there is little research addressing the method and process of PLNN. With the aforementioned context in mind, this study’s objectives were to describe the study participants’ demographic and surgical features regarding PLNN and assess the risk factors associated with these characteristics.

## Materials and methods

Study design

Patients diagnosed with AR were the subject of this cross-sectional study.

Study place and duration

This study was conducted among all patients with AR who were admitted and operated on at a tertiary care center in Tamaka, Kolar, over a five-year period (2018 to 2023).

Study population

During the study period, all patients with nasal allergies received PLNN surgery.

Sampling technique and sample size

All patients who met the inclusion criteria and underwent PLNN surgery during the study period were considered study participants by convenient sampling. Case sheets accessible at the medical record department were used to compile a list of 50 study subjects.

Ethical clearance

This study was conducted after receiving approval from the Institutional Ethics Committee of Sri Devaraj Urs Academy of Higher Education and Research (approval number: DMC/KLR/IEC/551 a/2022-23).

Inclusion and exclusion criteria

The study population was individuals aged between 18 and 65 years who were diagnosed with moderate-to-severe AR per Allergic Rhinitis and Its Impact on Asthma (ARIA) guidelines and who underwent PLNN surgery. Only patients with AR symptoms for at least two years (more than one hour per day, at least five days per week, and year-round) were included in the study. Patients with AR due to drug-induced or hormonal causes of rhinitis or nasal polyps were excluded. Patients with a prior history of nasal surgery and nose bleeding issues were also excluded.

Data collection and analysis

This study was done using secondary data. The data were extracted from case sheet histories, surgical notes, and postoperative notes. The data were entered in Microsoft Excel (Microsoft Corp., Redmond, WA, USA). SPSS version was used for data analysis (IBM Corp., Armonk, NY, USA). All quantitative variables were categorized by their means and standard deviations. All categorical variables were similarly described using frequency and percentage. The chi-square test was used to evaluate the relationship between risk variables and the surgical attributes of the research participants. A P-value of less than 0.05 was deemed statistically significant with a confidence range of 95%.

## Results

In total, 50 patients with AR underwent PLNN. The general and surgical characteristics of the study cohort are presented in Table 1. Nearly 54% of patients were younger than 30. Most study participants were male (60%). Most patients (46%) underwent PLNN alone, followed by PLNN plus septoplasty (36%). Three-fourths of the study population had all four nerves recognized, and only 4% of patients (only two nerves had been identified) had trouble locating the nerves.

**Table 1:** General and surgical characteristics of the study participants (n = 50). PLNN = posterior lateral nasal neurectomy; FESS = functional endoscopic sinus surgery

Variables		Frequency	Percent
Age (in years)		Mean = 30.4, standard deviation = 7.9	
Age group	Less than or equal to 30 years	27	54.0
	More than 30 years	23	46.0
Gender	Female	20	40.0
	Male	30	60.0
Intervention	Only PLNN	23	46.0
	PLNN with FESS	2	4.0
	PLNN with septoplasty	18	36.0
	PLNN with septoplasty and conchoplasty	3	6.0
	PLNN with septoplasty and FESS	3	6.0
	PLNN with septoplasty and inferior turbinectomy	1	2.0
Number of nerves identified during surgery	2	2	4.0
	3	10	20.0
	4	38	76.0

The duration of surgery and the amount of intraoperative blood loss are detailed in Table 2. The average intraoperative blood loss during PLNN surgery was 43.14 mL. The average length of the PLNN surgery was 62.1 minutes (approximately one hour).

**Table 2:** Distribution of the study population according to their duration of surgery and intraoperative blood loss (n = 50). PLNN = posterior lateral nasal neurectomy

	Approximate intraoperative blood loss during PLNN surgery	Duration of PLNN surgery (in minutes)
Mean	43.14	62.10
Median	43.00	55.00
Mode	48	30
Standard deviation	6.250	27.050
Minimum	33	30
Maximum	60	125
Interquartile range	38.0–48.0	40.0–80.0

The pre- and post-surgery hemoglobin values of the study participants are detailed in Table 3. The preoperative mean hemoglobin level was 13.11 g/dL, and the postoperative mean hemoglobin level was 12.78 g/dL. After surgery, the blood hemoglobin level fell by around 0.37 g/dL.

**Table 3:** Distribution of the study population according to their hemoglobin levels (n = 50). PLNN = posterior lateral nasal neurectomy; HB = hemoglobin

	Preoperative HB levels (g/dL)	Postoperative HB levels (g/dL)	Difference in HB levels (g/dL) before and after surgery
Mean	13.114	12.786	0.378
Median	13.300	13.000	0.300
Mode	14.0	11.2	0.2
Standard deviation	1.1395	1.1494	0.2957
Minimum	10.8	10.6	0.1
Maximum	14.9	14.5	1.3
Interquartile range	12.5–14.0	11.8–13.8	0.2–0.50

The relationship between the difference in hemoglobin levels before and after surgery and the duration of PLNN with gender is depicted in Table 4. The average duration of PLNN operations in females was 52.75 minutes, whereas the average duration in males was 68.33 minutes. According to an independent t-test (p = 0.045), this difference in mean was statistically significant.

**Table 4:** Association between risk factors according to the gender of the study population (n = 50). PLNN = posterior lateral nasal neurectomy; HB = hemoglobin

	Gender	N	Mean	Mean difference	P-value
Difference in HB levels (g/dL) before and after surgery	Female	20	0.430	0.0867	0.315
	Male	30	0.343		
Duration of PLNN surgery (in minutes)	Female	20	52.75	-15.583	0.045
	Male	30	68.33		

Table 5 demonstrates the relationship between risk variables and the identification of nerves among the study participants. Approximately 85% of female study participants were identified with four nerves during PLNN surgery compared to 70% of male study participants. According to the chi-square test (p = 0.018), this proportional difference was statistically significant.

**Table 5:** Association between risk factors with identification of nerves among the study population (n = 50).

Variables			Number of nerves identified			Chi-square value	P-value
			Two	Three	Four		
Age group	Less than or equal to 30 years	Count	2	3	22	3.790	0.092
		%	7.4%	11.1%	81.5%		
	More than 30 years	Count	0	7	16		
		%	0.0%	30.4%	69.6%		
Gender	Female	Count	2	1	17	6.654	0.018
		%	10.0%	5.0%	85.0%		
	Male	Count	0	9	21		
		%	0.0%	30.0%	70.0%		

## Discussion

It is possible for a transnasal endoscopic neurectomy of the posterior nasal nerve to be a life-changing operation in cases where AR drug treatment has been unsuccessful. Patients who suffer from severe rhinorrhea benefit substantially from the effectiveness of this surgical management as the supply of parasympathetic nerves is reduced. Sneezing is another bodily function that is dramatically altered when afferent sensory nerve fibers are severed. The potentially detrimental role of neurogenic inflammation in AR provides the basis for PLNN [12].

Additionally, a novel immunology-neurology bridge has been proposed, which would explain how the immune system and nerve impulses interact to begin and regulate inflammation. This would be a significant step forward in the field of immunology [13]. There is a possibility that neuropeptide production can alter the immunological and secretory functions of the nasal mucosa which can have a pathogenic role in AR [14]. The posterior nasal nerve is the source of most of the parasympathetic, sympathetic, and sensory input that is received by the nasal respiratory mucosa. Therefore, neurectomy appears to be effective in treating and managing AR symptoms by severing the nerve supply to the nasal mucosa [15]. The critical steps include bilateral sphenopalatine nerve blocks, transnasally or transorally via the greater palatine foramen; vertical incisions made behind the posterior fontanelle; and elevation of the mucoperiosteal flap. The sphenopalatine foramen and artery are identified. The posterior nasal nerve is located 4-5 mm inferior to the sphenopalatine artery and is resected or cauterized. The flaps are repositioned back into place. No postoperative nasal packing is required. The same procedure is performed on the opposite side for effective results.

This study found that the average age of the study population was 30.4 years. Most study participants were less than or equal to 30 years old (54%). In our study, most participants were male (60%). Zaghloul conducted a study in Egypt in 2020 and found that the average age of study participants was 28.02 ± 5.43 years and that the majority of patients were male (60.3%) [13].

This study revealed that around 46% of surgeries were independent PLNNs and that the majority of patients (76%) had four nerves removed during surgery. The average intraoperative blood loss during PLNN surgery was 43.14 mL. The mean hemoglobin levels before and after surgery were 13.11 and 12.78 g/dL, respectively. The average duration of the operation was 62 minutes. Zaghloul carried out a study in Egypt and found that there were no complications [13]. Our study had similar findings.

Ikeda et al. found that posterior nasal neurectomy inhibits the secretagogue motor and neurogenic inflammation generated by parasympathetic and sensory denervation [11]. Mori et al. [16] and Kobayashi et al. [17] also reported similar patient benefits following posterior nasal neurectomy. They concluded that selective excision of peripheral nerve branches can alleviate allergy symptoms. In their trial of PLNN using a harmonic scalpel on 20 patients, Kawamura et al. showed subjective improvement in nasal blockage, sneezing, and nasal discharge in 100%, 90%, and 75% of patients, respectively. No surgical complications were reported [18].

Study limitations

Because this was a cross-sectional study, the observed relationship should not be taken as evidence of causation. Although PLNN surgery was described in this article, it was not compared to any other treatment for nasal allergies. While the results are promising, they may have been improved by using a larger sample size and performing the research at multiple tertiary care centers. This study did not use any scales such as the rhino-conjunctivitis quality of life scale for assessing the quality of life before and after surgery. Moreover, the severity of the symptoms among the patients was not considered in this study.

## Conclusions

PLNN surgery provides consistent, robust results with long-term relief of allergic and vasomotor rhinitis-related nasal symptoms without the risk of complication. The majority of the participants in this study were male and younger. According to this study, nearly half of the patients underwent independent PLNNs, and nearly three-fourths had four nerves identified. The typical PLNN surgical procedure lasted one hour. Males and females required different amounts of time, with females requiring less time. During PLNN surgery, most females were identified with four nerves compared to male patients. It would be preferable to record the symptoms of AR before and at three, six, and 12 months after surgery using a numerical score.
